# Hereditary Syphilis in the Second Generation

**Published:** 1905-05-13

**Authors:** 


### HEREDITARY SYPHILIS IN THE SECOND GENERATION.

In May last year the French Society of Dermatology and Syphilography invited Dr. Edmond Fournier to supply an answer to the two following questions:—1. "Is there such a thing as active hereditary syphilis in the second generation? That is, can a syphilitic man transmit the disease in an active form to his grand-children?" 2. "Is there such a thing as dystrophic heredity in the second generation of syphilitics?" The answer is given in a recent book by Dr. Fournier, and is positive in either case. The data are drawn from an analysis of 116 cases, which supply the author with the two following conclusions of importance. (*a*) Hereditary syphilis in the second generation is competent to determine a mortality of about two-fifths of the descendants in that generation. (*b*) In about four-fifths of the cases the disease revealed itself in the shape of dystrophies similar in all respects to those of hereditary syphilis in the first generation.[1]

[1] La Médecine Moderne, April 12, 1905.

